# Case Report: An Unusual Cause for Recurrent Hemopericardium in a Patient With Dyspnea

**DOI:** 10.3389/fcvm.2021.755106

**Published:** 2021-11-15

**Authors:** Uyên Châu Nguyên, Astrid I. P. Vernemmen, Patrique Segers, Axel zur Hausen, Rob G. H. Driessen, Maurice J. H. M. Pluijmen, Sebastiaan C. A. M. Bekkers

**Affiliations:** ^1^Department of Cardiology, Maastricht University Medical Center, Maastricht, Netherlands; ^2^Department of Pathology, Maastricht University Medical Center, Maastricht, Netherlands; ^3^Department of Cardiothoracic Surgery, Maastricht University Medical Center, Maastricht, Netherlands; ^4^Department of Intensive Care Medicine, Maastricht University Medical Center, Maastricht, Netherlands

**Keywords:** hemopericardium, cardiac angiosarcoma, right heart sarcoma, dyspnea, pericardial effusion

## Abstract

The case concerns a female presenting with dyspnoea resulting from recurrent hemopericardium. Pericardiocentesis, coronary angiography, and extensive laboratory and imaging studies did not reveal the underlying etiology of the hemopericardium. Only after repeat and exploratory surgery, diffuse venous pericardial hemorrhages with localized thrombi typical of angiosarcoma were discovered.

## Introduction

Dyspnea is a common symptom in pericardial effusive disease. The etiology of pericardial effusion varies widely but often remains unknown. Primary cardiac tumors are extremely rare (1, 2) and often go undetected until a late stage of the disease.

The present case highlights the importance of considering angiosarcoma of the heart as a potential diagnosis in patients presenting with recurrent pericardial effusion, even in the absence of malignant cells in the pericardial fluid and absence of macroscopic lesions on non-invasive imaging.

## Case Description and Diagnostic Assessment

### Part 1

A 52-year-old woman presented at the emergency room (ER) in a regional hospital with progressive dyspnea, a dry cough and fatigue during several weeks despite taking oral antibiotics because of a suspected pneumonia. Three days prior to presentation she had experienced a severe dull thoracic and epigastric pain accompanied by nausea and vomiting that had resolved spontaneously. Besides taking ferrofumarate and cholecalciferol for iron-deficiency anemia and vitamin D deficiency, she had no previous medical history.

**Figure 1:**
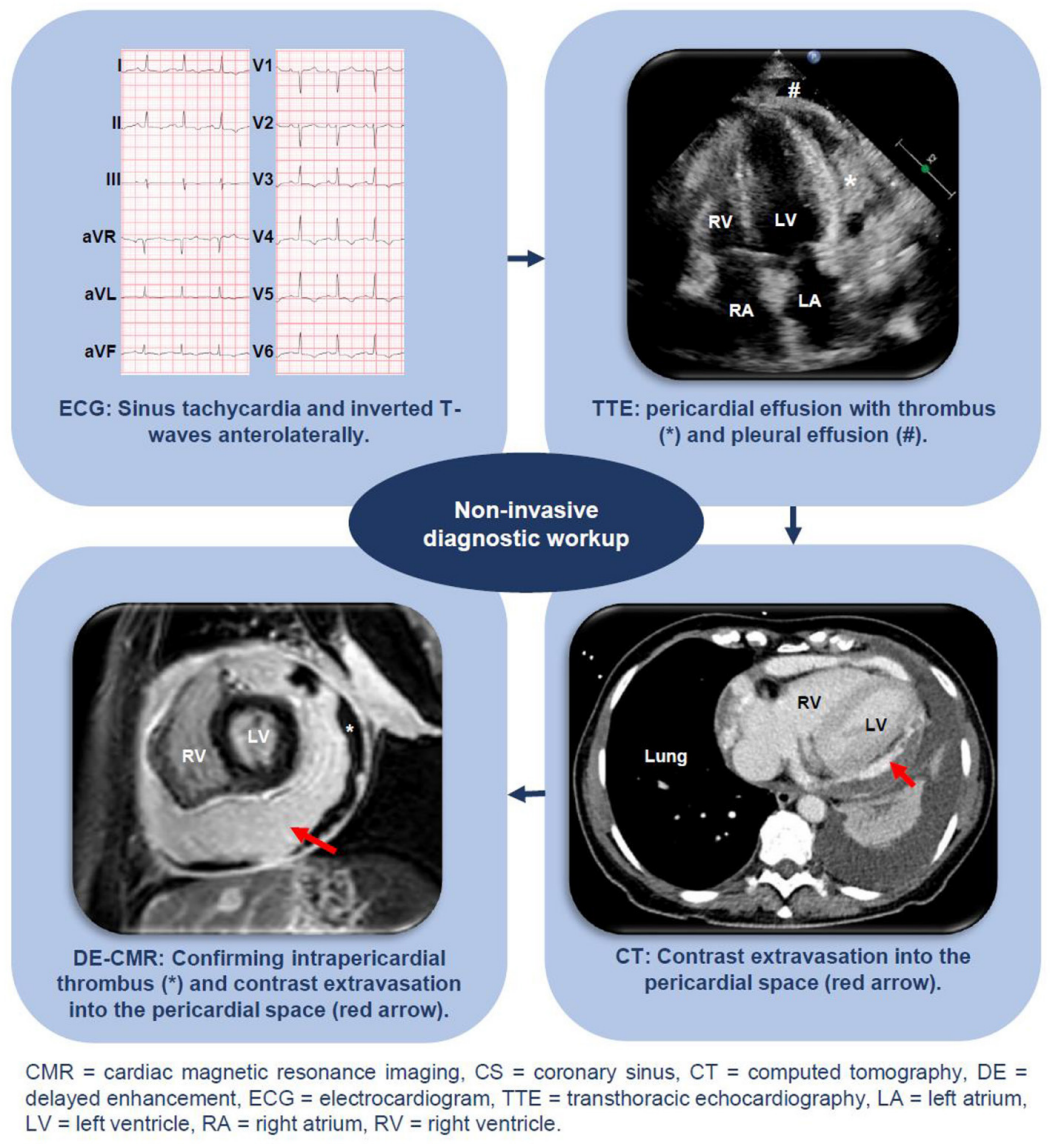
Non-invasive diagnostic workup including ECG, echocardiography, DE-CMR, and CT.

On presentation, physical examination revealed a regular tachycardia of 116 beats per minute (bpm), a blood pressure of 120/75 mmHg, an oxygen saturation of 100% while breathing ambient air, and a core temperature of 38.0°C (100.4°F). Cardiac, pulmonary, and abdominal examinations were unremarkable. There were no signs of deep venous thrombosis and the Wells-score was 4.5 (3). The electrocardiogram (ECG) showed sinus tachycardia, normal electrical heart axis and normal PR- and QRS-intervals, but inverted T-waves in both antero- and inferolateral leads (Figure 1). A low hemoglobin level (5 mmol/L), elevated c-reactive protein (102 mg/ml), troponin-I (1036 ng/L, cut-off is <20) and NT-pro-BNP (366 ng/L, normally <100) levels were the most prominent abnormal laboratory results.

Computed tomography angiography of the thorax (CTA) ruled out pulmonary embolism, but demonstrated pericardial and pleural effusion. Subsequent transthoracic echocardiography (TTE) confirmed circumferential pericardial effusion of ~4.5 cm with signs of haemodynamic compromise (dilated and poor collapsing inferior caval vein, early systolic right atrial (RA) collapse and >25% respiratory variation in peak mitral E-wave). Urgent evacuation of ~1 L of hemorrhagic fluid after pericardiocentesis immediately ameliorated symptoms. Investigation of this pericardial fluid did not reveal a tuberculous, bacterial, viral, or malignant etiology. A respiratory viral panel by polymerase chain reaction (influenzas A and B and respiratory syncytial virus) and a serum HIV antibody test were negative. After recurrent pericardial effusion was ruled out 1 week later, she was discharged home. While unconfirmed, a discharge diagnosis of post-infectious/post-viral pneumonia related pericardial and pleural effusion was made.

Already 5 days later, she presented again with progressive dyspnea, coughing, and nausea. A further decrease in hemoglobin (4.7 mmol/L) was noted and TTE showed recurrent circumferential pericardial effusion (~3 cm) but now a dense structure suggesting thrombus in the pericardial sac (Figure 1). Coronary angiography (CAG) was performed after which coronary artery disease including dissection could be ruled out.

### Part 2

She was transferred to a tertiary university center and received a blood transfusion. Additional viral serology (adenovirus, coxsackievirus, echovirus, borrelia burgdorferi, cytomegalovirus, Epstein-Barr virus, and parvovirus), immunological testing (systemic lupus erythematosus, rheumatoid arthritis, vasculitis, complement screening, and M-protein), a tuberculosis test and blood culture analyses were normal. To re-assess the possibility of a malignant cause, a repeat CTA of the thorax and abdomen was performed. While macroscopic malignancies could be ruled out, the scan showed contrast extravasation into the pericardial space, suggesting active bleeding. After a multidisciplinary consultation between cardiologists and cardiothoracic surgeons, and given the observation that the patient remained hemodynamically stable, it was decided that urgent surgery was not yet indicated and that there was still sufficient time for additional diagnostic workup. On cine cardiac magnetic resonance imaging (CMR), left (LV) and right ventricular (RV) systolic function were preserved and pleural and pericardial effusion confirmed. During contraction, the LV apex remained remarkably “fixed” to the pericardium (Supplementary Video 1). On the delayed enhancement (DE) images, the high intra-pericardial signal again suggested contrast-extravasation into the pericardial sac, whereas an extensive, hypo-enhanced circumferential layer against the inner parietal pericardium suggested thrombus (Figure 1). No intramyocardial abnormalities were observed.

It was concluded that ongoing, albeit slow, intra-pericardial bleeding was present. Since other diagnostic clues were missing at this time, an initial post-viral and subsequent recurrent traumatic or reactive pericardial effusion after pericardiocentesis were still considered causative and urgent surgery was yet indicated.

### Part 3

Since focal bleeding from a traumatic ventricular lesion after initial pericardiocentesis could not be excluded, it was decided to first perform a limited thoracotomy *via* a left-sided submammary incision. After evacuating 2.5 L hemorrhagic pericardial fluid with thrombi, careful inspection did not reveal a focal bleeding site. Because of persistent bleeding, the incision was extended further but again no focal bleeding source was discernable. Instead, multiple active venous hemorrhages on the entire epicardium were visible and hemostasis was attempted by placing multiple fibrin sealant patches Tachosil (Baxter healthcare cooperation, Illinois, USA). In addition, multiple biopsies of the pericardium and pericardial fluid and thrombi were sent for pathological examination.

The patient was subsequently transferred to the intensive care unit (ICU), but went into cardiogenic shock the following day. TTE showed recurrent pericardial effusion with a thrombus compressing the RA. A conventional emergency sternotomy was carried out. After thrombus removal, again diffuse hemorrhages were observed and a single bleeding focus in the RA was sutured.

**Figure 2:**
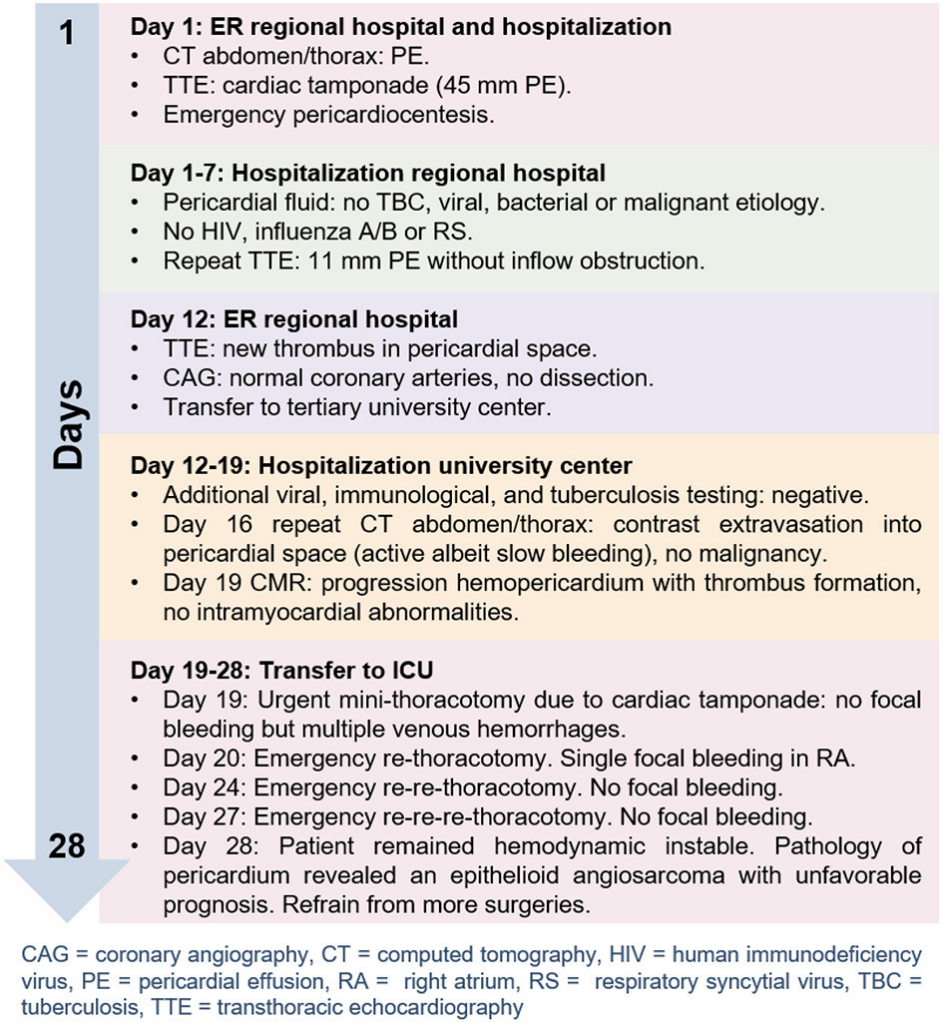
Figure showcasing the timeline of events.

Unfortunately, within 3 days she had to be operated two more times to relief recurrent cardiac tamponade and a left-sided hemothorax. Repeatedly, extensive diffuse venous epicardial hemorrhages were found that were difficult to manage, causing her to remain hemodynamically unstable in the ICU. After 9 days, pathology of the pericardium revealed an epithelioid angiosarcoma. Given the disease extent and unfavorable prognosis, it was decided to refrain from additional aggressive therapy. She died the same day, 28 days after the initial presentation. A timeline is showcased in Figure 2.

### Part 4

**Figure 3:**
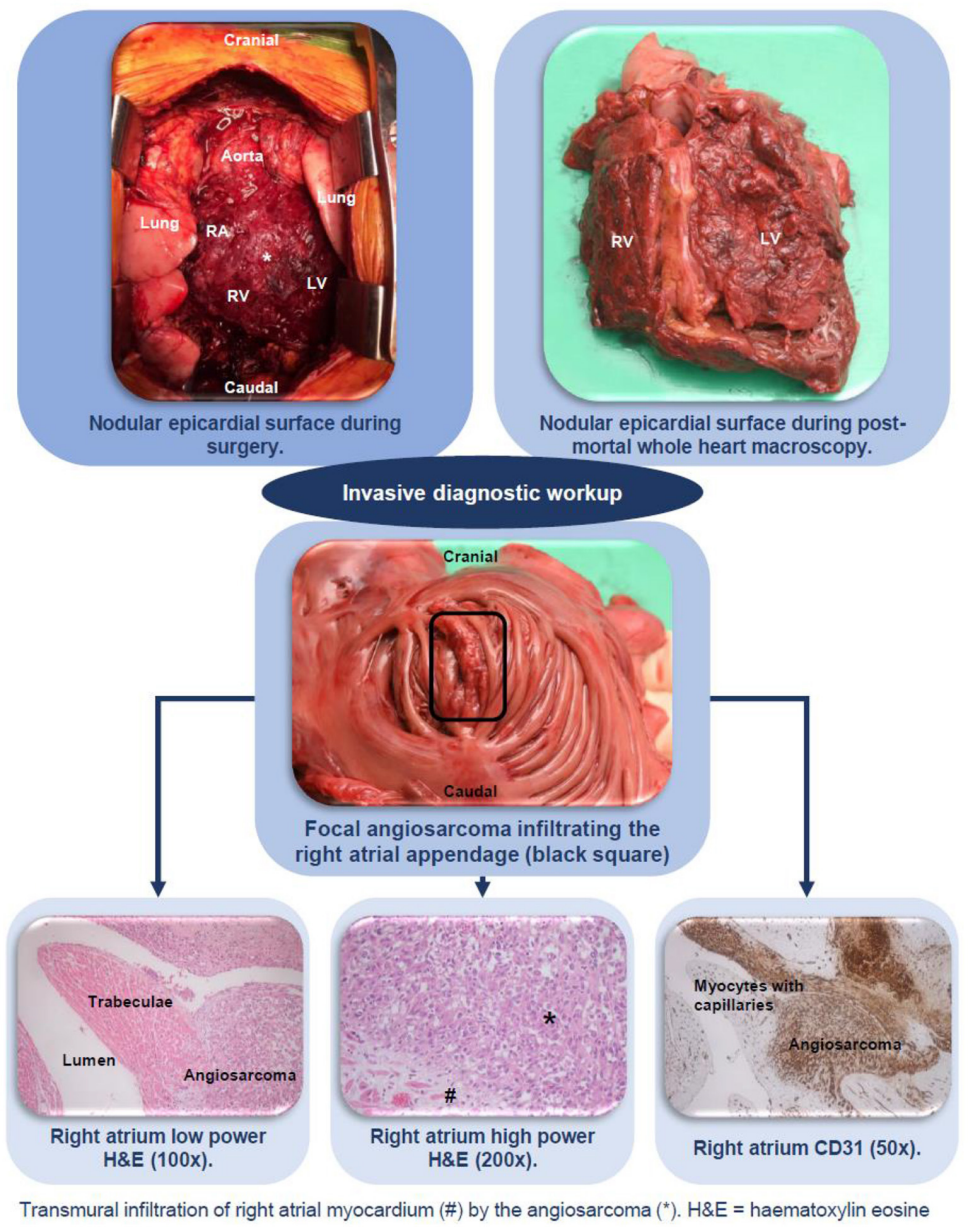
Invasive diagnostic workup including surgery and histopathology, confirming the diagnosis angiosarcoma of the heart.

After obtaining consent from relatives, gross autopsy revealed a diffusely thickened residual pericardium with an irregular surface with multiple thrombi and hemorrhages. The pericardium was extensively adhered to ribs, myocardium, ascending aorta, and aortic arch. A nodular epicardial surface was found with diffuse hemorrhages and thrombi. The heart was only minimally enlarged (419 [normal 233–403] g). Microscopy showed an atypical vascular proliferation with focal papillary structures consisting of large epithelioid cells with high amounts of eosinophilic cytoplasm. The nuclei of these epithelioid cells were pleomorphic and hyperchromatic with numerous, sometimes atypical, mitoses. Because these epithelioid cells stained positive for the immunohistochemical vascular markers ERG, CD31 and CD34 but not for CD68, HHV-8 and keratin markers CK AE1/AE3, CK 5/6, and CK7, a diagnosis of epithelioid angiosarcoma was made. The angiosarcoma was predominantly localized in the pericardium and epicardium without deeply infiltrating the myocardium leading to epicarditis and pericarditis. A single longitudinal thickened lesion was found at the RA appendage that had been surgically sutured. Microscopic evaluation of this lesion revealed transmural invasion of the angiosarcoma (Figure 3).

Additionally, lympho-vascular invasion with intracapillary tumor cells in the lungs (hemangiosis carcinomatosa) and focal invasion from the pericardium into the left lung was found, explaining the hemothorax.

## Discussion and Patient Perspective

Dyspnea, fatigue, thoracic pain, nausea, anorexia, and vomiting are common symptoms in pericardial effusive disease. The etiology of pericardial effusion often remains unknown but may be caused by malignancies that are mostly metastatic, complications of myocardial infarction, infections or iatrogenic. Differentiating between primary cardiac malignancies and other causes of pericardial effusion should be accomplished using non-invasive imaging, CAG, cytological and ideally histological investigation of pericardial effusion and tissue specimen, respectively (4).

Primary cardiac tumors are extremely rare with an autopsy incidence of <0.06% (1, 2) of which one quarter turns out to be malignant. Angiosarcoma is the most common primary cardiac malignancy (5) and has been first reported in 1934 by Barnes et al. (6). Cardiac angiosarcomas are aggressive tumors and are often fatal and metastases are found in the majority of patients (66–89%) at time of diagnosis (7).

Angiosarcomas are soft-tissue sarcomas of endothelial cell origin that may show features of vascular and/or lymphatic differentiation. The majority of angiosarcomas arise in the RA (8) in contrast to intimal or unclassified sarcomas, which typically arise from the left atrium or inter-atrial septum (9). Common sites of extension are the right coronary artery, myocardium of the LV and RV, superior and inferior caval vein, pericardium, and mediastinum.

Early diagnosis of cardiac angiosarcoma is difficult. Non-specific symptoms and disease rarity often prevent clinicians from inclusion in the initial differential diagnosis (2).

Previous cases have described angiosarcomas as irregulated lobulated masses extending into the pericardium and adjacent vessels and have demonstrated non-invasive imaging modalities to be useful (10).

CAG is generally recommended to rule out coronary artery disease and dissection (4). Pericardiocentesis is indicated in case of hemodynamic compromise (4), and to obtain material for cytological examination, although initially rarely yielding a conclusive diagnosis of angiosarcoma (11).

Histological investigation is often needed to finally diagnose angiosarcoma and tissue specimen can be obtained *via* thoracotomy or imaging-guided biopsy, although biopsies are often non-diagnostic while carrying considerable procedural risk. In the current European guidelines, a diagnostic epicardial/pericardial biopsy is a class IIA/B indication and only recommended after more than 3 weeks of illness (4).

Because cardiac angiosarcomas are rare, no evidence-based guidelines exist for its treatment. Complete surgical resection is indicated in case of a solitary lesion and if resection preserves cardiac integrity (12). Unfortunately, this is often not possible (12) because of its diffuse infiltrative nature. There is insufficient evidence that chemotherapy, radiotherapy or cardiac transplantation may improve survival (13, 14). As a result, prognosis of cardiac angiosarcomas remains poor with survival ranging from 6 to 9 months after diagnosis (13).

Cardiac angiosarcoma turned out to be a devil in disguise in our case and we were misled by the non-diagnostic results of the initial pericardiocentesis, CAG, and imaging results. Only after repeat and exploratory surgery, the diffuse venous pericardial hemorrhages with localized thrombi typical of angiosarcoma were discovered. At that time, the angiosarcoma was already disseminated with focal transmural invasion and only palliative options remained.

In retrospect, pathologically confirming angiosarcoma would have been possible several days earlier (day 12) if we had considered biopsy (either surgical or image-guided) immediately after transfer to our center. Whether this would have changed the outcome is doubtful, given that the tumor was already widely disseminated. Though, earlier diagnosis may have omitted repetitive surgery.

With the present case we would like to stress the importance of considering cardiac angiosarcoma as a potential diagnosis in recurrent pericardial effusion, even when malignant cells are absent in the pericardial fluid and macroscopic lesions on non-invasive imaging cannot (yet) be seen. An atypical clinical course of recurrent pericardial effusion may be typical for angiosarcoma of the heart.

## Data Availability Statement

The raw data supporting the conclusions of this article will be made available by the authors, without undue reservation.

## Ethics Statement

Ethical review and approval was not required for the study on human participants in accordance with the local legislation and institutional requirements. Written informed consent for participation was not required for this study in accordance with the national legislation and the institutional requirements.

## Author Contributions

UN and SB contributed to conception and design of the study and wrote the first drafts of the manuscript. AV, PS, AH, RD, and MP wrote sections of the manuscript. All authors contributed to manuscript revision, read, and approved the submitted version.

## Conflict of Interest

The authors declare that the research was conducted in the absence of any commercial or financial relationships that could be construed as a potential conflict of interest.

## Publisher's Note

All claims expressed in this article are solely those of the authors and do not necessarily represent those of their affiliated organizations, or those of the publisher, the editors and the reviewers. Any product that may be evaluated in this article, or claim that may be made by its manufacturer, is not guaranteed or endorsed by the publisher.

## Supplementary Material

Supplementary Video 1 Chamber cine CMR video.
